# The relationship between oxidative balance score and circadian syndrome: evidence from the NHANES 2005-2018

**DOI:** 10.3389/fendo.2024.1431223

**Published:** 2024-10-11

**Authors:** Lin Xie, Juan Li, Mingzhi Xu, Yahan Lei, Xushan Chen, Jiajia Xie

**Affiliations:** ^1^ The Seventh Clinical Medical College of Guangzhou University of Chinese Medicine, Shenzhen, Guangdong, China; ^2^ Shenzhen Bao’an Chinese Medicine Hospital, Guangzhou University of Chinese Medicine, Shenzhen, Guangdong, China

**Keywords:** oxidative balance score, circadian syndrome, NHANES, oxidative stress, circadian rhythm

## Abstract

**Background:**

The oxidative balance score (OBS) is a composite indicator that evaluates the balance between pro-oxidants and antioxidants in one’s diet and lifestyle. However, the relationship between OBS and circadian syndrome (CircS) has remained unexplored. This investigation aimed to determine a correlation between OBS and CircS.

**Methods:**

This population-based study examined 7,202 participants from the 2005 to 2018 National Health and Nutrition Examination Survey (NHANES), 1,433 of whom had CircS. We utilized weighted multivariate logistic regression, trend tests, subgroup analysis, and interaction tests to evaluate the correlation between OBS (total OBS, dietary OBS, and lifestyle OBS) and CircS. Restricted cubic splines (RCS) models and threshold effect analysis were used to explore nonlinear relationships.

**Results:**

Multivariate logistic regression analysis indicated that the protective factor for CircS was a high OBS level (total OBS: Odds ratio (OR) = 0.95, 95% Confidence interval (CI): 0.93-0.97; dietary OBS: OR = 0.98, 95% CI: 0.96-1.00; lifestyle OBS: OR = 0.65, 95% CI: 0.61-0.69). Compared to the quartile 1 group, OBS (total OBS, dietary OBS, and lifestyle OBS) was negatively and statistically significantly associated with the risk of developing Circs in the quartile 4 group (total OBS: OR = 0.47, 95% CI: 0.32-0.70; dietary OBS: OR = 0.69, 95% CI: 0.48-0.99; lifestyle OBS: OR = 0.07, 95% CI: 0.04-0.11). According to subgroup analysis and interaction tests, there was an interaction effect between the association of lifestyle OBS and CircS in terms of education level (*p* for interaction = 0.01). Furthermore, we observed a nonlinear negative relationship between lifestyle OBS and CircS prevalence, with inflection points at 6 (*p* for nonlinearity = 0.002).

**Conclusion:**

The results showed a substantial negative connection between OBS and CircS. Encouraging foods filled with antioxidants and antioxidant-rich lifestyles may reduce the risk of CircS.

## Introduction

Metabolic syndrome (MetS) is a set of cardiometabolic risk elements and comorbidities, including hypertension, abdominal obesity, insulin resistance, and dyslipidemia (1). It represents a substantial risk factor in the progression of atherosclerotic cardiovascular disease (2). MetS is becoming a global epidemic, affecting more than one in every five adults (3). Nevertheless, the common cause of MetS is still not fully comprehended (4). Circadian rhythm disturbance has become more prevalent in recent years due to factors like night lighting, social jet lag, meal timing, and shift or night shift work (5–8). An increasing amount of evidence supports that circadian rhythm disruption is strongly associated with MetS (9–11). In this situation, a new concept closely related to circadian rhythms, called circadian syndrome, has emerged (12).

The primary diagnostic criteria for CircS include elevated blood pressure, dyslipidemia, abdominal obesity, elevated blood glucose, short sleep, and depressive state (12). All of these symptoms are influenced by the circadian clock, implying that circadian rhythm disturbances could be a common cause (13–17). CircS affects around 40.8% of individuals in the United States (18). A comparable incidence of around 39% has been discovered among Chinese adults (19). Furthermore, studies have found that CircS is linked to a higher risk of stroke, kidney stones, overactive bladder syndrome, and chronic renal disease (20–23). The increased prevalence of CircS and its negative consequences emphasize the importance of early prevention and evaluation.

Oxidative stress is regarded as a predominant etiological factor in the occurrence of many diseases, and it causes an unbalanced between oxidant and antioxidant defenses in the body through the generation of reactive substances and redox signaling (24–26). Moderate antioxidant consumption has been demonstrated to modulate oxidative homeostasis and the levels of oxidative stress in the body (27, 28). In addition, Unhealthy lifestyles (e.g., smoking, alcohol misuse, and insufficient physical activity) have been recognized to disrupt redox homeostasis (29–31). OBS is a comprehensive metric that considers both pro-oxidants and antioxidants in one’s diet and lifestyle (32). Higher OBS reflects a lower level of oxidative stress (33). Some epidemiologic studies have identified a link between OBS and an increased rate of developing diabetes, cardiovascular disease, and stroke (34–36). However, no studies have examined whether OBS has affects CircS. As a result, We conducted a population-based study to assess the connection between OBS and CircS.

## Data and participants

**Figure 1:**
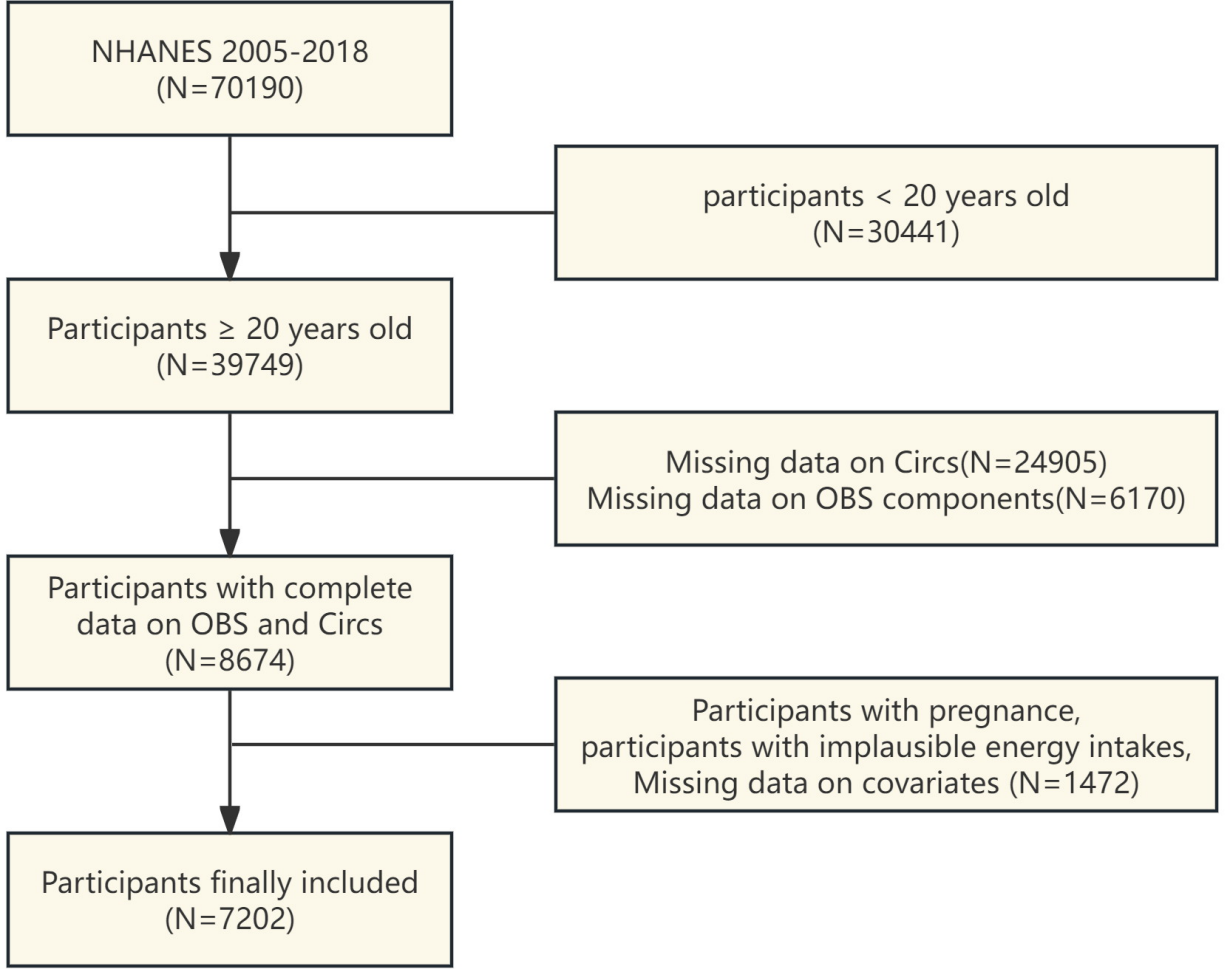
A flow chart of participants screening in NHANES 2005–2018.

NHANES is a program that conducts surveys every two years to estimate the nutritional status and health of children and adults in the United States. All participants grant their consent after being fully informed. Our analysis included data from seven successive NHANES cycles from 2005 to 2018, with a total of 70,190 people. We applied some exclusion standards: 1) Participants under the age of 20 (n = 30441); 2) missing CircS component data: waist circumference (WC, n = 3860), blood pressure (n = 1043), blood glucose (n = 18615), high-density lipoprotein cholesterol (HDL-C, n = 152), triglycerides (TG, n = 155), sleep (n = 34), and depression (n = 1046); 3) Missing OBS component data: inconsistent or missing dietary data (n=394), body mass index (BMI, n=35), serum cotinine (n = 7), and physical activity (n = 5734); 4) Covariates: serological markers (n = 47), low-density lipoprotein cholesterol (LDL-C, n = 146), education level (n = 5), PIR (n = 640), and marital status (n = 1); 5) Pregnancy (n = 153); 6) Unreliable energy intake (defined as men consuming < 800 kcal per day and > 4,200 kcal per day, and women consuming < 500 kcal per day and > 3,500 kcal per day) (n = 480). Our final study population consisted of 7,202 people (**Figure 1**).

## OBS and CircS definitions

The OBS is composed of 20 components, including 16 dietary and 4 lifestyle elements. The OBS was divided into tertiles according to gender differences, using previous studies’ allocation methodologies (37). Antioxidants were ranked from lowest to highest tertile, with 0, 1, and 2 scores. In contrast, ratings of 2 and 0 represent the lowest and highest pro-oxidant tertiles, respectively. Physical activity and body measures were allocated based on the metabolic equivalent (MET) score and BMI. Serum cotinine, a nicotine metabolite, was utilized as a biomarker to assess smoking. In terms of alcohol consumption, no alcohol intake was considered a nondrinker. Alcohol intake exceeds 30 g per day for males and 15 g per day for females were considered heavy drinkers. Alcohol consumption was categorized as nondrinkers, moderate drinkers, or heavy drinkers, with 0, 1, and 2 scores. **Table 1** shows the scheme for assigning OBS scores.

**Table 1:** Oxidative balance score assignment scheme.

OBS components	Property	Male			Female		
		0	1	2	0	1	2
Dietary fiber (g/d)	A	<13.6	13.6-21.25	≥21.25	<11.75	11.75-17.7	≥17.7
Carotene (RE/d)	A	<106.96	106.96-382.79	≥382.79	<121.96	121.96-439.79	≥439.79
Riboflavin (mg/d)	A	<1.77	1.77-2.54	≥2.54	<1.41	1.41-2	≥2
Niacin (mg/d)	A	<22.83	22.83-32.13	≥32.13	<16.49	16.49-23.32	≥23.32
Total folate (mcg/d)	A	<335.5	335.5-499	≥499	<264	264-393	≥393
Vitamin B6 (mg/d)	A	<1.75	1.75-2.55	≥2.55	<1.32	1.32-1.92	≥1.92
Vitamin B12 (mcg/d)	A	<3.6	3.6-6.13	≥6.13	<2.63	2.63-4.57	≥4.57
Vitamin C (mg/d)	A	<44.2	44.2-103.9	≥103.9	<43.5	43.5-93.25	≥93.25
Vitamin E (ATE) (mg/d)	A	<6.04	6.04-9.39	≥9.39	<5.2	5.2-8.11	≥8.11
Calcium (mg/d)	A	<756	756-1140	≥1140	<629.5	629.5-931.5	≥931.5
Magnesium (mg/d)	A	<260.5	260.5-365	≥365	<217.5	217.5-296	≥296
Zinc (mg/d)	A	<9.77	9.77-14	≥14	<7.38	7.38-10.32	≥10.32
Copper (mg/d)	A	<1.06	1.06-1.51	≥1.51	<0.89	0.89-1.25	≥1.25
Selenium (mcg/d)	A	<102.75	102.75-142.6	≥142.6	<74.7	74.7-105.3	≥105.3
Iron (mg/d)	P	≥18.23	12.72-18.23	<12.72	≥14.25	9.88-14.25	<9.88
Total fat (g/d)	P	≥98.25	67.74-98.25	<67.74	≥75.04	51.71-75.04	<51.71
Physical activity (MET-minute/week)	A	<720	720-1988	≥1988	<540	540-1440	≥1440
BMI(kg/m2)	P	≥29.52	25.3-29.52	<25.3	≥30.52	24.7-30.52	<24.7
Cotinine (ng/mL)	P	≥0.5	0.02-0.5	<0.02	≥0.07	0.01-0.07	<0.01
Alcohol (g/d)	P	≥30	0-30	<0	≥15	0-15	<0

OBS, oxidative balance score; A, antioxidant; P, pro-oxidant; RE, retinol equivalent; ATE, alpha-tocopherol equivalent; MET, metabolic equivalent; BMI, body mass index.

CircS comprises elements of metabolic syndrome, short sleep, and depressive state. The diagnosis relies on the subsequent standards: (1) WC equal to or exceeding 102 cm in males and 88 cm in females, (2) TG equal to or exceeding 1.7 mmol/L or previous recommendation by a physician to take lipid-lowering medications, (3) HDL-C levels below 1.0 mmol/L in males and below 1.3 mmol/L in females or previous recommendation by a physician to take lipid-lowering medications, (4) Systolic blood pressure (SBP) equal to or greater than 135 mmHg or diastolic blood pressure (DBP) equal to or greater than 85 mmHg, or use of antihypertensive drugs, (5) Fasting blood glucose (FBG) equal to or exceeding 100 mg/dL or use of hypoglycemic drugs and insulin, (6) sleeping less than 6 hours, and (7) Depression scores equal to or greater than 9 on the Health Questionnaire. Participants diagnosed with CircS exhibited four or more of these seven components.

## Covariates

Based on previous research (38, 39), we included a range of covariates that may be associated with CircS. Covariates consisted of age (20–39, 40–65, or ≥65 years), gender (male or female), race (Mexican American, Non-Hispanic White, Non-Hispanic Black others), marital status (married or partnered, never married, widowed or divorced, separated), education attainment (below high school, high school, above high school), and PIR (≤1.3, 1.3–3.5, or >3.5). Energy intake, LDL–C, serum cholesterol, uric acid (UA), creatinine (Cr), aspartate aminotransferase (AST), alanine aminotransferase (ALT), and gamma-glutamyl transferase (GGT).

## Statistical analyses

All data were analyzed with the NHANES complex weighted sampling design. The OBS quartile stratified the study population’s baseline characteristics (Q1: < 25th percentile; Q2: 25–50th percentile; Q3: 50–75th percentile; Q4: ≥ 75th percentile). Weighted means and standard deviations are utilized to calculate constant variables, whereas weighted percentages are employed to calculate categorical variables. We utilized chi-square and t-tests to analyze differences within the OBS quartile classifications. The correlation between OBS and CircS was investigated in four distinct models using multivariable logistic regression. Model 1 was free of any covariate components. Model 2 included modifications for age, gender, and race. Model 3 included modifications for age, gender, race, marital status, education attainment, PIR, and energy intake. Model 4 was additionally adjusted for serum cholesterol, LDL-C, ALT, AST, Cr, GGT, and UA based on Model 3. Dietary OBS and lifestyle OBS were calculated according to gender differences to examine the robustness of the correlation between OBS and CircS. Then, stratified analyses and interaction tests were performed based on age, gender, race, marital status, education attainment, and PIR. In the end, RCS analysis and threshold effect analysis were applied to investigate the non-linear relationship between OBS and the risk of CircS. All the analyses were conducted employing R (version 4.3.2), with a statistically significant *p*-value < 0.05.

## Results

### General characteristics of the participants

7702 individuals were enrolled in our study, with a mean age of 46.31 ± 16.65 years. Among these, 50.12% were males, and the prevalence of CircS was found to be 26.92%. OBS was classified into four categories based on gender differences: Q1, Q2, Q3, and Q4. The corresponding sample sizes were as follows: 2016, 1865, 1593, and 1728. The range of scores for the following groups: Q1 had a range of <15 points, Q2 had a range of 15-20 points, Q3 had a range of 21-25 points, and Q4 had a range of >25 points. The proportion of people diagnosed with CircS declined as the quartiles increased. People in the top quartile of OBS were inclined to be 40-65 years old, female, non-Hispanic white, have an education level above high school, have a PIR greater than 3.5, be married or live with a partner, consume more energy, and have lower levels of GGT, AST, ALT, and UA. Furthermore, in the top quartile of OBS, the CircS seven diagnostic components were the least prevalent. **Table 2** depicts the population’s baseline characteristics and variables categorized by the OBS quartile.

**Table 2:** Weighted demographic characteristics of all participants.

Characteristic	Overall, N = 7202 (100%)	Q1, N = 2016 (23%)	Q2, N = 1865 (25%)	Q3, N = 1593 (23%)	Q4, N = 1728 (29%)	p-value
Age(years)	46.31 ± 16.65	46.14 ± 17.76	47.15 ± 17.30	45.48 ± 16.03	46.38 ± 15.56	0.2
years(%)						0.005
20-39	2,805(40.30%)	775(41.77%)	700(39.11%)	634(41.61%)	696(39.11%)	
40-65	2,967(42.92%)	779(38.83%)	771(41.36%)	681(44.44%)	736(46.37%)	
≥65	1,430(16.78%)	462(19.40%)	394(19.53%)	278(13.94%)	296(14.52%)	
Gender(%)						0.7
Male	3,656(50.12%)	998(48.74%)	962(50.65%)	821(51.39%)	875(49.76%)	
Female	3,546(49.88%)	1,018(51.26%)	903(49.35%)	772(48.61%)	853(50.24%)	
Race(%)						<0.001
Mexican American	969(6.93%)	285(8.07%)	256(7.30%)	196(6.52%)	232(6.03%)	
Non-Hispanic White	3,309(70.80%)	773(62.53%)	838(69.30%)	752(72.04%)	946(77.79%)	
Non-Hispanic Black	1,429(9.48%)	557(15.70%)	384(10.56%)	293(8.35%)	195(4.42%)	
Others	1,495(12.79%)	401(13.70%)	387(12.84%)	352(13.10%)	355(11.77%)	
Education level(%)						<0.001
Below high school	1,203(10.45%)	452(15.67%)	327(10.26%)	222(9.66%)	202(7.03%)	
High school	1,493(19.73%)	533(28.22%)	415(22.22%)	299(17.57%)	246(12.40%)	
Above high school	4,506(69.83%)	1,031(56.12%)	1,123(67.51%)	1,072(72.76%)	1,280(80.57%)	
PIR group(%)						<0.001
≤1.3	1,875(17.63%)	664(25.26%)	497(18.73%)	378(15.71%)	336(12.04%)	
1.3-3.5	2,619(32.49%)	819(38.84%)	707(33.96%)	551(31.87%)	542(26.57%)	
>3.5	2,708(49.88%)	533(35.90%)	661(47.30%)	664(52.42%)	850(61.40%)	
Marital status(%)						<0.001
Married/Living with partner	4,337(63.12%)	1,094(54.26%)	1,142(63.60%)	980(66.05%)	1,121(67.49%)	
Never married	1,458(20.14%)	451(24.43%)	360(20.11%)	320(18.04%)	327(18.39%)	
Widowed/Divorced/Separated	1,407(16.74%)	471(21.31%)	363(16.28%)	293(15.91%)	280(14.12%)	
Elevated BP(%)						<0.001
No	4,485(66.45%)	1,135(60.37%)	1,122(63.43%)	1,037(70.10%)	1,191(71.08%)	
Yes	2,717(33.55%)	881(39.63%)	743(36.57%)	556(29.90%)	537(28.92%)	
Elevated FPG(%)						<0.001
No	3,588(52.55%)	875(46.36%)	937(52.17%)	809(51.98%)	967(58.33%)	
Yes	3,614(47.45%)	1,141(53.64%)	928(47.83%)	784(48.02%)	761(41.67%)	
Elevated WC(%)						<0.001
No	3,517(49.63%)	786(38.72%)	879(46.34%)	798(49.92%)	1,054(61.13%)	
Yes	3,685(50.37%)	1,230(61.28%)	986(53.66%)	795(50.08%)	674(38.87%)	
Low HDL-C(%)						<0.001
No	5,512(78.69%)	1,424(73.76%)	1,412(74.66%)	1,251(80.12%)	1,425(85.08%)	
Yes	1,690(21.31%)	592(26.24%)	453(25.34%)	342(19.88%)	303(14.92%)	
Elevated TG(%)						0.001
No	2,036(46.94%)	498(41.96%)	516(43.26%)	463(47.95%)	559(53.56%)	
Yes	2,623(53.06%)	816(58.04%)	724(56.74%)	548(52.05%)	535(46.44%)	
Short sleep(%)						<0.001
No	6,322(90.63%)	1,683(86.63%)	1,639(90.60%)	1,420(91.01%)	1,580(93.58%)	
Yes	880(9.37%)	333(13.37%)	226(9.40%)	173(8.99%)	148(6.42%)	
Depression symptoms(%)						<0.001
No	6,776(94.88%)	1,851(91.48%)	1,749(94.43%)	1,508(95.37%)	1,668(97.61%)	
Yes	426(5.12%)	165(8.52%)	116(5.57%)	85(4.63%)	60(2.39%)	
Energy intake (kcal)	2,094.44 ± 757.15	1,580.51 ± 580.28	1,930.38 ± 639.34	2,248.48 ± 698.56	2,530.35 ± 729.80	<0.001
BMI (kg/m2)	28.11 ± 6.23	29.74 ± 6.60	28.52 ± 6.33	28.00 ± 5.89	26.51 ± 5.67	<0.001
WC (cm)	96.79 ± 15.64	100.52 ± 16.30	97.93 ± 15.74	96.52 ± 15.02	92.98 ± 14.62	<0.001
SBP(mmHg)	120.45 ± 16.22	122.59 ± 17.37	121.53 ± 16.85	119.33 ± 15.19	118.68 ± 15.21	<0.001
DBP(mmHg)	69.83 ± 11.58	69.56 ± 12.51	70.14 ± 12.26	69.87 ± 10.84	69.74 ± 10.72	0.8
HDL-C (mmol/L)	1.45 ± 0.42	1.38 ± 0.39	1.42 ± 0.42	1.45 ± 0.40	1.53 ± 0.44	<0.001
LDL-C (mmol/L)	2.96 ± 0.91	2.95 ± 0.95	2.97 ± 0.92	3.00 ± 0.90	2.92 ± 0.89	0.2
TG (mmol/L)	1.28 ± 0.72	1.35 ± 0.76	1.32 ± 0.72	1.26 ± 0.72	1.20 ± 0.69	<0.001
Cholesterol (mmol/L)	5.02 ± 1.06	4.96 ± 1.10	5.02 ± 1.09	5.06 ± 1.01	5.05 ± 1.03	0.2
GGT (U/L)	26.26 ± 38.22	28.07 ± 30.64	27.90 ± 57.11	25.79 ± 32.99	23.72 ± 24.11	0.002
Cr(umol/L)	78.06 ± 27.24	80.43 ± 39.97	79.13 ± 28.78	76.63 ± 18.75	76.35 ± 16.71	0.062
AST (U/L)	25.12 ± 14.13	24.43 ± 11.87	24.87 ± 13.02	25.43 ± 15.60	25.63 ± 15.44	<0.001
ALT (U/L)	25.04 ± 16.99	25.05 ± 17.09	24.70 ± 17.10	25.40 ± 18.40	25.04 ± 15.59	0.033
UA (umol/L)	323.99 ± 79.66	333.60 ± 82.62	329.22 ± 81.28	321.26 ± 77.87	313.80 ± 75.85	<0.001
Dietary OBS	16.80 ± 6.66	7.69 ± 2.49	14.22 ± 2.31	19.27 ± 2.07	24.44 ± 2.34	<0.001
Lifestyle OBS	3.68 ± 1.75	2.91 ± 1.65	3.41 ± 1.71	3.72 ± 1.63	4.49 ± 1.62	<0.001
CircS(%)						<0.001
No	3,226(73.08%)	774(63.89%)	865(70.22%)	726(75.89%)	861(80.92%)	
Yes	1,433(26.92%)	540(36.11%)	375(29.78%)	285(24.11%)	233(19.08%)	

Circs, Circadian syndrome; PIR, Proverty income ratio; FPG,Fasting plasma glucose; BMI, Body Mass Index; WC, Waist circumference; SBP, Systolic blood pressure;DBP, Diastolic blood pressure; HDL-C, High-Density Lipoprotein cholesterol; LDL-C, Low-Density Lipoprotein cholesterol; TG, Triglycerides; GGT, Gamma-glutamyl transferase; Cr, Creatinine; AST, Aspartate aminotransferase; ALT, Alanine aminotransferase; UA, Uric acid.

Wilcoxon rank-sum test for complex survey samples; chi-squared test with Rao & Scott’s second-order correction.

mean ± standard deviation (SD) for continuous; n (%) for categorical.

### Relationship between total OBS and CircS

As shown in **Table 3**, weighted logistic regression models were employed to investigate the correlation between total OBS and CircS. When total OBS was measured as a continuous variable, there was a significant correlation between higher OBS and lower prevalence of CircS. The negative correlation between total OBS and CircS was also present even after adjustment for covariates (model 4), implying a 5% decrease in the prevalence of CircS for each unit increase in total OBS. (OR = 0.95, 95% CI: 0.93-0.97, *p* < 0.001). We further converted OBS into a quartile variable for sensitivity analysis. With individuals in the Q1 group as a reference, the reduction in the risk of developing CircS was statistically significant for those in groups Q3 and Q4 (*p* < 0.05). In the fully adjusted model (model 4), the ratio of individuals in groups Q3 and Q4 developing CircS was 0.61 (95% CI: 0.44- 0.84) and 0.47 (95% CI: 0.32 - 0.70), respectively.

**Table 3:** Relationship between total OBS and CircS.

Model 1				Model 2			Model 3			Model 4		
Characteristic	OR	95% CI	p-value	OR	95% CI	p-value	OR	95% CI	p-value	OR	95% CI	p-value
Continuous	0.95	0.93, 0.96	<0.001	0.95	0.94, 0.96	<0.001	0.94	0.92, 0.95	<0.001	0.95	0.93, 0.97	<0.001
OBS (quartile)												
Q1		Ref			Ref			Ref			Ref	
Q2	0.75	0.58, 0.97	0.027	0.74	0.57, 0.95	0.020	0.72	0.55, 0.94	0.016	0.80	0.60, 1.07	0.14
Q3	0.56	0.44, 0.72	<0.001	0.57	0.44, 0.74	<0.001	0.52	0.39, 0.69	<0.001	0.61	0.44, 0.84	0.003
Q4	0.42	0.31, 0.56	<0.001	0.43	0.32, 0.58	<0.001	0.36	0.25, 0.53	<0.001	0.47	0.32, 0.70	<0.001
P for trend		<0.001			<0.001			<0.001			<0.001	

OR, Odds Ratio; CI, Confidence Interval; OBS, oxidative balance score.

Model 1, No covariates were adjusted.

Model 2, adjusted for sex, age, race.

Model 3, adjusted for sex, age, race, marital status, education attainment, PIR, and energy intake.

Model 4, adjusted for sex, age, race, education attainment, marital status, PIR, energy intake, LDL-C,cholesterol, UA, Cr, AST, ALT and GGT.

### Association of dietary OBS and lifestyle OBS with CircS

As shown in **Table 4**, dietary OBS and lifestyle OBS were both negatively associated with CircS in Model 4. For each unit increase in dietary OBS, the risk of CircS would be reduced by 2% (OR = 0.98, 95% CI: 0.96-1.00, *p* = 0.02). Lifestyle OBS seemed more closely related to the risk of CircS than dietary OBS. For each unit increase in lifestyle OBS, the risk of CircS would be reduced by 35% (OR = 0.65, 95% CI: 0.61-0.69, *p* < 0.001). We transformed dietary OBS and lifestyle OBS into categorical variables. In all four models, there was a statistically significant negative correlation between lifestyle OBS and CircS in the other three groups, using the Q1 group as a reference (*p* < 0.001). There was also a negative correlation observed between dietary OBS and CircS. This relationship was statistically significant in the Q4 group (model 1: OR = 0.56, 95% CI: 0.42-0.74, p < 0.001; model 2: OR = 0.57, 95% CI: 0.43-0.77, p < 0.001; model 3: OR = 0.56, 95% CI: 0.40-0.79, p = 0.001; model 4: OR = 0.69, 95% CI: 0.48-0.99, p = 0.046). According to interaction tests, no interaction effect was identified between dietary OBS and lifestyle OBS (*p* for interaction = 0.133).

**Table 4:** Association of dietary OBS and lifestyle OBS with CircS.

	Model 1			Model 2			Model 3			Model 4		
Characteristic	OR	95% CI	p-value	OR	95% CI	p-value	OR	95% CI	p-value	OR	95% CI	p-value
Dietary OBS												
Continuous	0.97	0.96, 0.98	<0.001	0.97	0.96, 0.98	<0.001	0.97	0.95, 0.98	<0.001	0.98	0.96, 1.00	0.020
OBS (quartile)												
Q1		Ref			Ref			Ref			Ref	
Q2	0.79	0.60, 1.02	0.074	0.77	0.59, 1.01	0.055	0.78	0.60, 1.01	0.058	0.85	0.63, 1.14	0.3
Q3	0.76	0.60, 0.95	0.019	0.75	0.59, 0.96	0.025	0.75	0.57, 0.99	0.041	0.87	0.64, 1.17	0.4
Q4	0.56	0.42, 0.74	<0.001	0.57	0.43, 0.77	<0.001	0.56	0.40, 0.79	0.001	0.69	0.48, 0.99	0.046
P for trend		<0.001			<0.001			0.002			0.04	
Lifestyle OBS												
Continuous	0.60	0.57, 0.64	<0.001	0.61	0.57, 0.64	<0.001	0.61	0.58, 0.65	<0.001	0.65	0.61, 0.69	<0.001
OBS (quartile)												
Q1		Ref			Ref			Ref			Ref	
Q2	0.50	0.39, 0.63	<0.001	0.51	0.40, 0.65	<0.001	0.52	0.41, 0.66	<0.001	0.54	0.42, 0.69	<0.001
Q3	0.25	0.20, 0.30	<0.001	0.25	0.20, 0.31	<0.001	0.26	0.21, 0.32	<0.001	0.31	0.25, 0.39	<0.001
Q4	0.05	0.03, 0.08	<0.001	0.05	0.03, 0.09	<0.001	0.06	0.04, 0.09	<0.001	0.07	0.04, 0.11	<0.001
P for trend		<0.001			<0.001			<0.001			<0.001	

OR, Odds Ratio; CI, Confidence Interval; OBS, Oxidative Balance Score.

Model 1, No covariates were adjusted.

Model 2, adjusted for sex, age, race.

Model 3, adjusted for sex, age, race, marital status, education attainment, PIR, and energy intake.

Model 4, adjusted for sex, age, race, education attainment, marital status, PIR, energy intake, LDL-C,cholesterol, UA, Cr, AST, ALT and GGT.

### Subgroup analysis and interaction tests for total OBS and CircS

**Figure 2:**
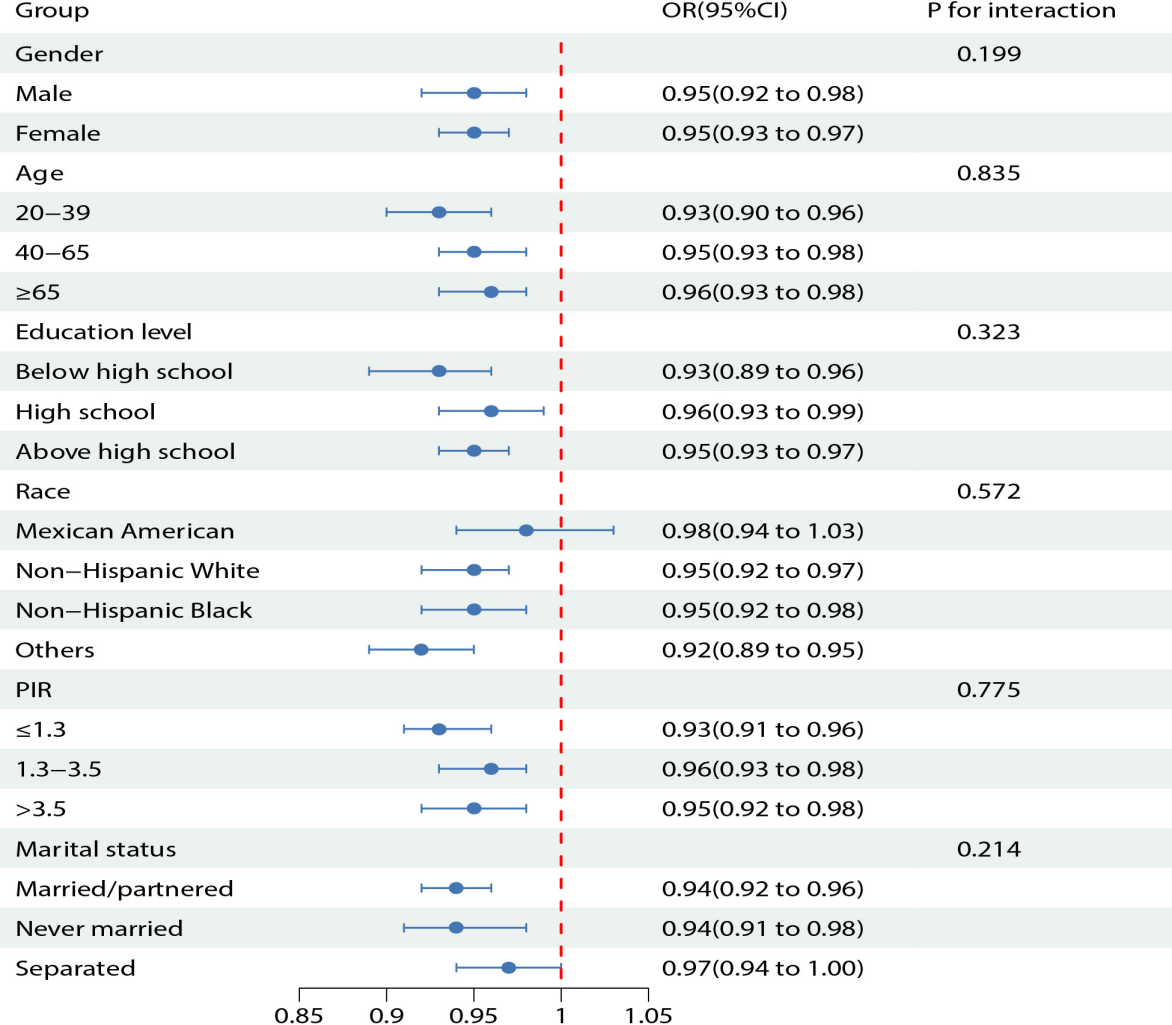
Subgroup analysis and interaction tests for the association between total OBS and CircS.

We applied subgroup analysis and interaction tests according to age, gender, race, education attainment, and marital status. The outcomes of subgroup analyses revealed that the association between total OBS and CircS varied among groups. Participants exhibited substantial differences in subgroups divided by age, gender, education attainment, PIR, and marital status (*P* < 0.05). A negative relationship was also observed among the Mexican American population in the ethnicity stratification, but this relationship was not statistically significant (*p* = 0.4). Interaction tests found that total OBS did not correlate with age, gender, race, education attainment, PIR, and marital status. **Figure 2** displays the association between CircS and total OBS in various subgroups in model 4.

### Subgroup analysis and interaction tests of dietary OBS and lifestyle OBS with CircS

Subgroup analysis exploring the connection between dietary OBS and CircS identified differences in the following categories: females, aged 20-39, below high school, individuals of other ethnic backgrounds, married or cohabiting individuals, and those with a PIR of less than or equal to. Subgroup analysis exploring the correlation between lifestyle OBS and CircS revealed that all subgroups observed differences that are statistically significant (*p* < 0.001). The interaction test proved an interaction effect between lifestyle OBS and education attainment (*p* for interaction = 0.01). Higher levels of education were associated with a more significant correlation between lifestyle OBS and CircS (OR = 0.61, 95% CI: 0.56-0.66, *p* < 0.001). The subgroup analysis and interaction tests for OBS (total OBS, dietary OBS, and lifestyle OBS) and CircS are described in detail in **Supplementary Tables 1**-**3**.

### Restricted cubic spline analysis

**Figure 3:**
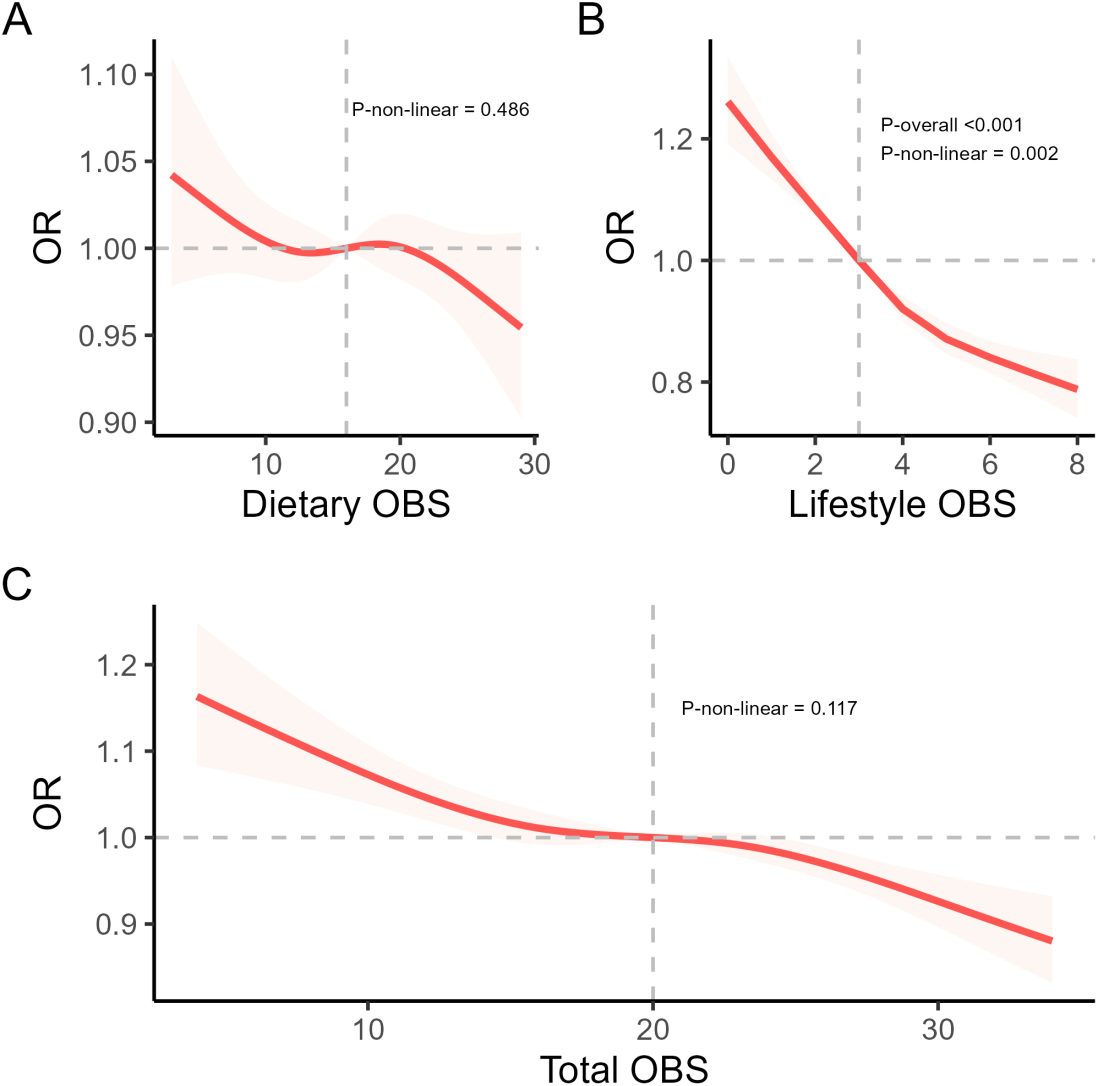
The RCS analysis of OBS and CircS. **(A)** a linear negative correlation between dietary OBS and CircS. **(B)** a non-linear negative correlation between lifestyle OBS and CircS. **(C)** a linear negative correlation between total OBS and CircS.

Adjusting for all confounders, RCS curves were utilized to further explore the connection between OBS (total OBS, dietary OBS, and lifestyle OBS) and CircS. We found a linear correlation between OBS(total OBS and dietary OBS) and CircS (total OBS: *p* = 0.117; dietary OBS: *p* = 0.486). A nonlinear negative connection was identified between lifestyle OBS and CircS (*p* = 0.002). **Figure 3** displays the results of the analysis of RCS.

Furthermore, the inflection point for this nonlinear relationship between lifestyle OBS and CircS was found to be at point 6. Following this, we conducted a threshold effect analysis centered on this inflection point. On the left side of the turning point, the effect size was 0.341 (95% CI: 0.148 -0.653), and on the right side of the turning point, the effect size was 0.684 (95% CI: 0.651- 0.717). **Table 5** describes the results of the threshold effect analysis.

**Table 5:** Threshold effect analysis of lifestyle OBS on CircS by the two-piecewise linear regression.

Inflection point	Adjusted OR (95% CI)	*P*-value
< 6	0.341(0.148-0.653)	0.004
≥6	0.684(0.651-0.717)	<0.001
Log-likelihood ratio	0.038	

Adjusted for sex, age, race, education attainment, marital status, PIR, energy intake, LDL-C, cholesterol, UA, Cr, AST, ALT and GGT. OBS, oxidative balance score; CircS, circadian syndrome; OR, odds ratio; 95% CI, 95% confidence interval.

## Discussion

As circadian rhythm disruption becomes more common with changing lifestyles in our society, early intervention for CircS is necessary (40). This study eventually involved 7202 patients from the NHANES 2005-2018 cohort, with 1433 having CircS. Patients with CircS have lower OBS (total OBS, dietary OBS, and lifestyle OBS) than normal individuals. This negative connection persisted even after controlling for all variables. Although the effect estimates for the connection between OBS and CircS varied by subgroup, their correlation remained negative across all categories. The interaction test results revealed a pronounced connection between lifestyle OBS and CircS in people with education attainment levels above high school. In the RCS, we discovered that total OBS and dietary OBS were linearly and adversely linked to CircS, while lifestyle OBS was nonlinearly and negatively associated. In addition, lifestyle OBS was significantly negatively associated with CircS risk in an overall trend both before and after the inflection point. We identified the inflection point at 6.

To our knowledge, this study is the first to evaluate the correlation between OBS and CircS. CircS is composed of MetS, short sleep duration, and depressive state. Numerous studies have revealed a substantial link between oxidative stress and these factors. A population-based study by Zhixiao Xu et al. exploring the correlation between OBS and MetS among adult Americans showed that higher OBS was related to a lower incidence of MetS and all-cause mortality (38). Another cross-sectional study found a significant correlation between OBS and MetS along with its components (41). In addition to cardiometabolic risk profiles, oxidative stress was similarly associated with sleep quality and depression. Populations with higher OBS have fewer sleep disturbances and have longer sleep duration (42). A randomized controlled design suggests that lowering anti-oxidative stress improves depression scores in patients with major depressive disorder (43). Overall, these epidemiologic studies imply that elevated levels of oxidative stress may increase the likelihood of developing CircS. As expected, we discovered that higher OBS was negatively linked with the development of CircS.

Dietary and lifestyle changes raise the risk of circadian disturbances. Many studies have found significant correlations between the components of OBS and CircS. A cross-sectional study found that consuming vegetables, whole grains, oils, nuts, and seeds helped to reduce the incidence of CircS (44). Keizo Kaneko and his colleagues demonstrated that obesity interferes with the circadian expression of crucial clock genes in the central nervous system (45). Alcohol misuse disrupts circadian rhythms (46). Grigsby, K et al. reported that chronic alcoholism affected circadian gene expression (47). Research has demonstrated that nicotine can induce phase alterations in circadian rhythms in rats (48). Fuentes-Cano, Martin A. et al. discovered that the circadian locomotion and learning efficiency rhythms of juvenile rodents are influenced by perinatal nicotine exposure (49). In addition, Camerbiru et al. found that physical exercise synchronized the circadian system and prevented the development of CircS in indoor fat sand rats (50).

Given that excellent eating habits and lifestyle contribute to regulating the circadian rhythm, our work suggests non-pharmacological prophylactic treatment for the affected population. We can help patients regulate their biological clocks by promoting healthy eating habits and lifestyles, such as increasing physical activity, adhering to regular schedules, limiting high-calorie foods, and reducing sedentary time. Furthermore, our findings alert medical professionals to the lifestyle behaviors of patients with circadian abnormalities.

Interestingly, interaction tests indicated that the negative correlation between lifestyle OBS and CircS was more significant among participants with high levels of schooling. Some studies have revealed that people with less education are more prone to lead unhealthy lifestyles (51, 52). Individuals with higher levels of education have higher levels of self-perceived health and a greater ability to engage in healthier behaviors (53). Additionally, people with higher levels of education engage in physical activity for longer periods and have access to better sports facilities (54). These all contribute to health-promoting behaviors.

The relationship between OBS and CircS is uncertain. Metabolic alterations in CircS are strongly associated with disruption of circadian rhythms, and we thus hypothesize that oxidative stress may have an impact on the regulatory control of circadian rhythms. Oxidative stress is triggered by the hyperproduction of damaging reactive oxygen species (ROS) and reactive nitrogen species (RNS) (55). Oxidative stress arises as the balance of ROS, nitric oxide, and antioxidants is interrupted, causing DNA, lipids, proteins, and cellular structural damage (56). Cellular redox status regulates the biological clock, and rhythmic ROS generates redox oscillations that give feedback to the cellular clock machinery (57). Schmalen, Ira, et al. discovered that maintaining adequate oxidative homeostasis governs the complexes generated by cyclins and cryptochromes, which are key components of circadian regulation (58). Second, melatonin, a neurobiological hormone generated by the pineal gland, regulates circadian rhythms (59). Melatonin deficiency can lead to prolonged wakefulness, brain injury, and metabolic disturbance (60–64). In an animal model, the researchers discovered that persistent stress causes decreased levels of pineal melatonin (65). It was found that myocardial infarction-induced oxidative stress caused an increase in free radicals, which in turn decreased plasma melatonin levels (66). In addition, Koh, Kyunghee, et al. found that increased oxidative stress disrupted the sleep-wake cycle (66). Such research is currently limited, but preliminary findings warrant further investigation.

This study emphasizes the significance of oxidative stress levels for the development of CircS and enriches existing studies. However, there are certain constraints to this study. First, the design of our research was cross-sectional, which may be subject to other confounding factors and the inability to determine causality. Second, our study population was primarily from the United States. Therefore, we were unable to generalize the results to different populations. Finally, it has been demonstrated that shift work, exposure to light, and noise all produce circadian disruptions (67). We cannot rule out all of these confounding factors. Further comprehensive studies are needed to elucidate the causal relationship between OBS and CircS prevalence.

## Conclusion

The results showed a substantial negative connection between OBS and CircS. Encouraging foods filled with antioxidants and antioxidant-rich lifestyles may decrease the risk of CircS. Nevertheless, additional studies are required to investigate the potential mechanisms between OBS and CircS.

## Data Availability

The original contributions presented in the study are included in the article/**Supplementary Material**. Further inquiries can be directed to the corresponding author.
