# Supplementary material for: The relationship between oxidative balance score and circadian syndrome: evidence from the NHANES 2005-2018
Source: Front Endocrinol (Lausanne). 2024 Oct 11;15:1431223. doi: 10.3389/fendo.2024.1431223 (PMC11512453; doi:10.3389/fendo.2024.1431223)
Supplement: Supplementary file 1 [file Table1.docx]

The Relationship between Oxidative Balance Score and Circadian Syndrome: evidence from the NHANES 2005-2018

Lin Xie^1^, Juan Li^1^, Mingzhi Xu^1^ , Yahan Lei^1^, Xushan Chen^2^ and Jiajia Xie*^2^

^1^The seventh clinical medical college of Guangzhou University of Chinese Medicine, Shenzhen, Guangdong Province, 518000 China

^2^Shenzhen Bao’an Chinese Medicine Hospital, Guangzhou University of Chinese Medicine, Shenzhen, Guangdong Province, 518000 China

Corresponding author: Jiajia Xie,

*Correspondence: [xiejiajiabazyy@163.com](mailto:xiejiajiabazyy@163.com)

Supplemental Table S1 Subgroup analysis and interaction test of the association between Total OBS and CircS.

| Group | Characteristic | OR | 95% CI | *p*-value | *p* for interaction |
| --- | --- | --- | --- | --- | --- |
| Gender |  |  |  |  | 0.199 |
|  | Male | 0.95 | 0.92, 0.98 | <0.001 |  |
|  | Female | 0.95 | 0.93, 0.97 | <0.001 |  |
| Age |  |  |  |  | 0.835 |
|  | 20-39 | 0.93 | 0.90, 0.96 | <0.001 |  |
|  | 40-65 | 0.95 | 0.93, 0.98 | <0.001 |  |
|  | ≥65 | 0.96 | 0.93, 0.98 | 0.003 |  |
| Education level |  |  |  |  | 0.323 |
|  | Below high school | 0.93 | 0.89, 0.96 | <0.001 |  |
|  | High school | 0.96 | 0.93, 0.99 | 0.016 |  |
|  | Above high school | 0.95 | 0.93, 0.97 | <0.001 |  |
| Race |  |  |  |  | 0.572 |
|  | Mexican American | 0.98 | 0.94, 1.03 | 0.4 |  |
|  | Non-Hispanic White | 0.95 | 0.92, 0.97 | <0.001 |  |
|  | Non-Hispanic Black | 0.95 | 0.92, 0.98 | 0.004 |  |
|  | Others | 0.92 | 0.89, 0.95 | <0.001 |  |
| PIR |  |  |  |  | 0.775 |
|  | ≤1.3 | 0.93 | 0.91, 0.96 | <0.001 |  |
|  | 1.3-3.5 | 0.96 | 0.93, 0.98 | <0.001 |  |
|  | > 3.5 | 0.95 | 0.92, 0.98 | 0.003 |  |
| Marital status |  |  |  |  | 0.214 |
|  | Married/Living with partner | 0.94 | 0.92, 0.96 | <0.001 |  |
|  | Never married | 0.94 | 0.91, 0.98 | 0.003 |  |
|  | Widowed/Divorced/Separated | 0.97 | 0.94, 1.00 | 0.026 |  |

Abbreviations: Circs, Circadian syndrome; OBS, Oxidative balance score; OR, Odds ratio; CI, Confidence interval.

Supplemental Table S2 Subgroup analysis and interaction test of the association between Dietary OBS and CircS.

| Group | Characteristic | OR | 95% CI | *p*-value | *p* for interaction |
| --- | --- | --- | --- | --- | --- |
| Gender |  |  |  |  | 0.07 |
|  | Male | 0.98 | 0.95, 1.01 | 0.2 |  |
|  | Female | 0.97 | 0.94, 0.99 | 0.009 |  |
| Age |  |  |  |  | 0.917 |
|  | 20-39 | 0.96 | 0.92, 0.99 | 0.022 |  |
|  | 40-65 | 0.98 | 0.95, 1.00 | 0.095 |  |
|  | ≥65 | 0.99 | 0.95, 1.02 | 0.4 |  |
| Education level |  |  |  |  | 0.487 |
|  | Below high school | 0.95 | 0.91, 0.99 | 0.009 |  |
|  | High school | 0.98 | 0.95, 1.01 | 0.2 |  |
|  | Above high school | 0.98 | 0.96, 1.01 | 0.2 |  |
| Race |  |  |  |  | 0.801 |
|  | Mexican American | 1.01 | 0.96, 1.05 | 0.8 |  |
|  | Non-Hispanic White | 0.98 | 0.95, 1.01 | 0.11 |  |
|  | Non-Hispanic Black | 0.97 | 0.94, 1.01 | 0.11 |  |
|  | Others | 0.95 | 0.92, 0.99 | 0.006 |  |
| PIR |  |  |  |  | 0.504 |
|  | ≤1.3 | 0.96 | 0.93, 0.99 | 0.005 |  |
|  | 1.3-3.5 | 0.97 | 0.95, 1.00 | 0.07 |  |
|  | > 3.5 | 0.99 | 0.96, 1.02 | 0.5 |  |
| Marital status |  |  |  |  | 0.121 |
|  | Married/Living with partner | 0.97 | 0.95, 0.99 | 0.007 |  |
|  | Never married | 0.98 | 0.95, 1.02 | 0.4 |  |
|  | Widowed/Divorced/Separated | 0.99 | 0.96, 1.02 | 0.7 |  |

Abbreviations: Circs, Circadian syndrome; OBS, Oxidative balance score; OR, Odds ratio; CI, Confidence interval, PIR, Poverty income ratio.

Supplemental Table S3 Subgroup analysis and interaction test of the association between Lifestyle OBS and CircS.

| Group | Characteristic | OR | 95% CI | *p*-value | *p* for interaction |
| --- | --- | --- | --- | --- | --- |
| Gender |  |  |  |  | 0.759 |
|  | Male | 0.64 | 0.58, 0.71 | **<0.001** |  |
|  | Female | 0.67 | 0.60, 0.75 | **<0.001** |  |
| Age |  |  |  |  | 0.839 |
|  | 20-39 | 0.63 | 0.54, 0.72 | **<0.001** |  |
|  | 40-65 | 0.65 | 0.59, 0.72 | **<0.001** |  |
|  | ≥65 | 0.65 | 0.57, 0.74 | **<0.001** |  |
| Education level |  |  |  |  | **0.01** |
|  | Below high school | 0.67 | 0.58, 0.78 | **<0.001** |  |
|  | High school | 0.76 | 0.66, 0.87 | **<0.001** |  |
|  | Above high school | 0.61 | 0.56, 0.66 | **<0.001** |  |
| Race |  |  |  |  | 0.11 |
|  | Mexican American | 0.73 | 0.61, 0.88 | **0.001** |  |
|  | Non-Hispanic White | 0.64 | 0.58, 0.70 | **<0.001** |  |
|  | Non-Hispanic Black | 0.73 | 0.64, 0.83 | **<0.001** |  |
|  | Others | 0.58 | 0.51, 0.67 | **<0.001** |  |
| PIR |  |  |  |  | 0.215 |
|  | ≤1.3 | 0.64 | 0.56, 0.73 | **<0.001** |  |
|  | 1.3-3.5 | 0.7 | 0.64, 0.76 | **<0.001** |  |
|  | > 3.5 | 0.62 | 0.56, 0.69 | **<0.001** |  |
| Marital status |  |  |  |  | 0.645 |
|  | Married/Living with partner | 0.64 | 0.59, 0.69 | **<0.001** |  |
|  | Never married | 0.58 | 0.50, 0.67 | **<0.001** |  |
|  | Widowed/Divorced/Separated | 0.69 | 0.60, 0.79 | **<0.001** |  |

Abbreviations: Circs, Circadian syndrome; OBS, Oxidative balance score; OR, Odds ratio; CI, Confidence interval.
